# Emodin and aloe-emodin, two potential molecules in regulating cell migration of skin cells through the MAP kinase pathway and affecting *Caenorhabditis elegans* thermotolerance

**DOI:** 10.1186/s12860-023-00486-1

**Published:** 2023-07-25

**Authors:** Aysenur Gunaydin-Akyildiz, Rabia Sare Yanikoglu, Meltem Gulec, Gulbahar Ozge Alim-Toraman, Ebru Didem Kuran, Sezen Atasoy, Abdullah Olgun, Gulacti Topcu

**Affiliations:** 1 Faculty of Pharmacy, Department of Pharmaceutical Toxicology, Bezmialem Vakif University, Istanbul, 34093 Turkey; 2 Faculty of Pharmacy, Department of Biochemistry, Bezmialem Vakif University, Istanbul, 34093 Turkey; 3 Faculty of Pharmacy, Istinye University, Istanbul, 34010 Turkey; 4 Faculty of Pharmacy, Department of Pharmacognosy, Bezmialem Vakif University, Istanbul, 34093 Turkey; 5 Faculty of Pharmacy, Department of Pharmaceutical Chemistry, Bezmialem Vakif University, Istanbul, 34093 Turkey; 6 Faculty of Medicine, İstinye University, Istanbul, 34010 Turkey

**Keywords:** Emodin, Aloe-emodin, Molecular docking, MAP kinase, JNK, P38, ERK, *C. elegans*, Thermotolerance

## Abstract

**Background:**

Emodin and aloe-emodin are two anthraquinones having positive effects in wound healing. However, their mechanism of action of wound healing is not fully understood. The MAP kinase family, which plays an active role in wound healing, is a well-characterized large family of serine/threonine kinases and regulates processes such as proliferation, oncogenesis, differentiation, and inflammation in the cell. The aim of this study is to comparatively elucidate the mechanisms of action of emodin and aloe-emodin, which are potential agents in wound healing.

**Methods:**

The mechanism of the effects of emodin and aloe-emodin on cell viability and cell migration was examined using the human skin fibroblast (CCD-1079Sk) cell line. The gene expression levels of the MAP kinases (JNK, P38, ERK) in the skin fibroblast cells along with a molecular docking study analyzing their interaction potential were evaluated. Furthermore, the molecules’ effects on the lifespan of *Caenorhabditis elegans* were studied.

**Results:**

Emodin and aloe-emodin inhibited the ATP content of the cells in a concentration dependent manner and accelerated cell migration at the lower concentrations while inhibiting cell migration in the higher concentration treatment groups. The expressions of *JNK* and *P38* were upregulated at the low concentrations and downregulated at the higher concentrations. The molecular docking studies of the molecules gave high docking scores indicating their interaction potential with JNK and P38. *C. elegans* lifespan under heat stress was observed longer after 75 µM emodin and was significantly reduced after 150 µM aloe-emodin treatment.

**Conclusion:**

Aloe-emodin was found to be more potent on cell viability, cell migration, gene expression levels of the MAP kinases in healthy fibroblastic skin cells, and on the lifespan of *C. elegans*. This study reveals the functional effects and the biological factors that interact in the wound healing process of emodin and aloe-emodin, and give a possible treatment alternative to shorten the duration of wound care.

## Introduction

The skin represents the largest organ of the human body and its main function is protection from external stresses. This protective feature can be compromised when the skin deteriorates as a result of mechanical attacks. Wound healing is an evolutionarily conserved physiological process that aims to preserve the integrity of the skin and restore the skin barrier after trauma. The normal wound healing process includes three consecutive and overlapping phases: the hemostasis/inflammatory phase, the proliferative phase, and the remodeling phase. Each stage of wound healing is essential for a normal healing progress because the interruption of any stage delays healing and can cause various skin pathologies, including failure of recovery or chronic ulceration [1, 2]. Various specialized cells such as platelets, macrophages, fibroblasts, epithelial and endothelial cells are involved in the wound healing process. These cells interact with each other and with the extracellular matrix. Fibroblasts, in particular, play an active role in the proliferation phase of the healing process [3].

The anthraquinone derivatives emodin (1,3,8-trihydroxy-6-methyl-anthraquinone) and aloe-emodin (1,8-dihydroxy-3-(hydroxymethyl)-anthraquinone) are found in many plants traditionally used in wound healing such as *Aloe vera, Polygonum cuspidatum, Rheum officinale* [4] which are among the secondary metabolites of the species. The aim of this study is to comparatively elucidate the mechanisms of action of emodin and aloe-emodin, which are potential agents in wound healing.

**Fig. 1:**
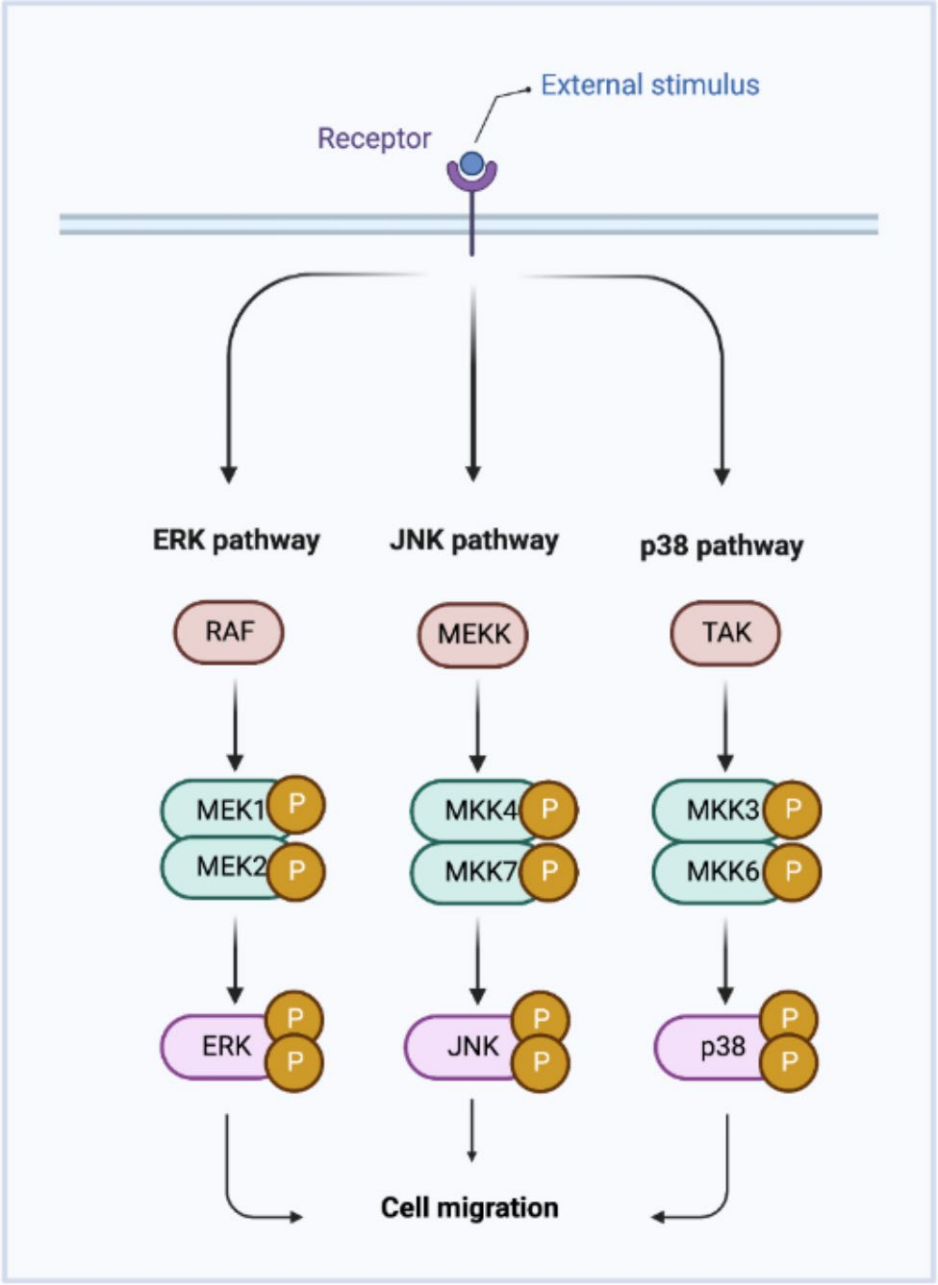
**MAP kinase signaling pathways related with cell migration.** Created with BioRender.com

The MAP kinase family, which plays an active role in wound healing, is a well-characterized large family of serine/threonine kinases and regulates processes such as proliferation, oncogenesis, differentiation, and inflammation in the cell. The MAP kinase family, which is examined in three major groups; Jun N-terminus kinase (JNK) pathway, extracellular signal regulatory kinases (Erk1/2) pathway and P38 group protein kinase pathway, controls important cellular functions which are also the basis of wound healing [5–7]. Figure 1. shows the diagram of these MAP kinase signaling pathways.

JNK activity plays an important role in the migration of fibroblasts in wound healing experiments [8]. Erk1/2 is one of the main molecules of the MAPK signaling pathway activated through the three-component protein kinase cascade Raf → MEK → Erk which is critical in promoting angiogenesis during wound healing [6, 9]. P38 is known to play a role in apoptosis, cell differentiation and cell migration [5].

In traditional folk medicine, herbs are widely used as natural molecules and derivatives from plants are found to be effective and safe. The extracts prepared from medicinal plants and the molecules/derivatives isolated from these extracts create an important potential for scientific research considering the developing phytochemical research and technology Although the use of natural products in wound healing is common, studies investigating the mechanisms of action are insufficient [4]. The elucidation of the mechanisms of action of various effective wound healing natural compounds may offer alternatives for the treatment of very persistent wounds such as diabetic neuropathic lower extremity ulcers [6].

In this study, the gene expression levels of the MAP kinase (*JNK, P38, ERK*) signaling pathway were evaluated after emodin and aloe-emodin treatment in CCD-1079Sk human skin fibroblast cells. Additionally, a molecular docking study was conducted analyzing the interaction potential of emodin and aloe-emodin with the MAP kinases. Molecular docking is a method that allows us to determine the interactions of a protein -whose crystal structure has been reported in the literature- and our original molecules under optimal conditions. The in vitro wound healing properties of the natural molecules emodin and aloe-emodin in the CCD-1079Sk skin fibroblast cell line was investigated. The effects of emodin and aloe-emodin on intracellular ATP content were examined. Understanding the mechanisms that regulate cell migration and proliferation of dermal fibroblast cells by the action of a natural compound will be useful in designing new treatments to regulate fibrosis and wound contraction by accelerating the wound healing process [10]. Furthermore, the molecules’ effects on the lifespan of *Caenorhabditis elegans (C.elegans)* under heat stress (thermotolerance) were studied to obtain in vivo preclinical data.

## Materials and methods

### Cell Culture

Human skin fibroblasts CCD-1079Sk (CRL-2097™) were purchased from American Type Culture Collection (ATCC, VA, USA), and cultured in DMEM-F12 medium supplemented with 10% fetal bovine serum and 1% penicillin–streptomycin (Gibco, USA). Sub-culturing was done every 2–3 days.

### Treatment

Emodin and aloe-emodin were purchased from Sigma-Aldrich (MO, USA) and dissolved in DMSO. The studied concentrations were chosen according to the cytotoxicity assay which was done in the range of 0-1000 µM with the ATP assay. According to the IC_50_ values, for the evaluation of the gene expression levels 25 µM, 12.5 µM, and 6.25 µM were selected for emodin; 10 µM, 5 µM, and 2.5 µM for aloe-emodin. The control group was the solvent containing the same percentage of DMSO (0.1%) with the treatment groups.

### ATP Bioluminescence Assay

ATP content of the cells was determined with recording the bioluminescence using CellTiter-Glo® 2.0 (Promega, USA). The luminescent signal is proportional to the ATP content of the cells which is an indicator of cell viability [11]. Opaque white 96-well plates were used. CCD-1079Sk cells were seeded 10^4^/well and treatment was conducted after a 24 h incubation. Following 24 h treatment with emodin and aloe-emodin, the cells were shaken for 2 min with CellTiter-Glo® 2.0 Reagent which was added the same amount with the culture medium present in each well. After 10 min incubation in room temperature, the luminescence was recorded using a BioTek Synergy H1 (Epoch, Germany) microplate reader. The results are given as a percentage relative to the solvent control group.

### In vitro wound healing (scratch) assay

For evaluating the migration of the CCD-1079Sk cells. The CytoSelect™ 24-well Wound Healing Assay Kit (Cell Biolabs Inc, USA) was used. The inserts were placed inside every well of the 24 well plate using a sterile forceps with their “wound field” aligned in the same direction. 500 µL of medium containing 2 × 10^5^ cells was added to the gaps of the inserts. After 24 h incubation in a cell culture incubator overnight for the cells to provide a monolayer, the inserts were removed, and the images of each well were taken before treatment. After taking the images, emodin (6.25 µM, 12.5 µM, and 25 µM) and aloe-emodin (2.5 µM, 5 µM, and 10 µM) treatments were done for 24 h and the images of the same area of the wounds were taken. For calculating the wound healing percentage, the program ImageJ (National Institutes of Health, USA) was used, and comparison was done between the treatment groups with the vehicle control group.

### Evaluation of gene expression levels

Gene expressions of *JNK*, *P38*, and *ERK* were evaluated by reverse transcription polymerase chain reaction (RT-PCR) and *Beta actin (βactin)* was used as house-keeping gene. Total RNA extraction was done according to the manufacturer’s instructions of Quick RNA Mini Prep Kit (Zymo Research). cDNA synthesis was done using SensiFAST™ cDNA Synthesis Kit (Bioline). RT-PCR was performed with the SensiFAST™ SYBR No-ROX Kit (Bioline). Gene expression levels were calculated by the 2^-ΔΔCT^ method [12]. The studied primer sequences can be found in Table 1.

**Table 1:** Primer sequences for determination of gene expression levels

Gene	Forward Primer	Reverse Primer	Melting Temperature (ºC)
***JNK***	GACGCCTTATGTAGTGACTCGC	TCCTGGAAAGAGGATTTTGTGGC	62.1
***P38***	GAGCGTTACCAGAACCTGTCTC	AGTAACCGCAGTTCTCTGTAGGT	62.2
***ERK***	TGGCAAGCACTACCTGGATCAG	GCAGAGACTGTAGGTAGTTTCGG	62
***βactin***	CATGTACGTTGCTATCCAGGC	CTCCTTAATGTCACGCACGAT	62

### Molecular docking study

The docking study was carried out using the Schrödinger Software Suite (Maestro Schrödinger Release 2022-2: Meastro, Schrödinger, LLC, New York, NY, 2021). The crystal structure of N-terminus kinase-1 (JNK1) complexed with its inhibitor quercetagetin at 2.70 A ° resolution and the crystal structure of p38alpha complexed with pyrazolobenzothiazine inhibitor COXH11 (9Y5) at 2.50 A ° (PDB ID: 3V3V and 5OMH, respectively) was retrieved from the RCSB Protein Data Bank [13, 14]. The crystal structures of the enzymes were prepared using the multi-step Protein Preparation Wizard Module of Schrödinger Software Suite (Schrödinger Release 2022-2: Protein Preparation Wizard; Epik, Schrödinger, LLC, New York, NY, 2021; Impact, Schrödinger, LLC, New York, NY; Prime, Schrödinger, LLC, New York, NY, 2021) [15]. The retrieved crystal structure was optimized by removing water molecules, heteroatoms, and co-factors. The hydrogens, missing atoms, bonds, and charges were computed and corrected through Maestro. The studied compounds (emodin and aloe-emodin) and the co-crystallized ligands (quercetagetin and 9Y5) were also prepared and optimized (generating various tautomers and ring conformations, assigning bond orders, and stereochemistries) using the LigPrep module of Schrödinger Software Suite (Schrödinger Release 2022-2: LigPrep, Schrödinger, LLC, NY, 2021). All the conformations generated were minimized using the OPLS4 force field. The receptor grid was generated around the co-crystalized ligand of the enzyme to specify the binding site, where the grid box size was determined with the receptor Grid Generation implemented in Glide (Schrödinger Release 2022-2: Glide, Schrödinger, LLC, NY, 2021) [16–18]. The Glide docking was performed in the Standard Precision (SP) mode.

### *C. elegans* Survival (Thermotolerance) assay under heat stress

*C. elegans* used in this experiment were obtained from the *Caenorhabditis* Genetics Center CGC. The type used was the wild N2 strain and was maintained in the Nematode Growth Medium (NGM). One-liter volume of NGM was prepared as follows: First agar 17 g, NaCl 3 g, and peptone 2.5 g were mixed and autoclaved at 121 °C for 15 min. After the flask cooled down to 55 °C; 1 M MgSO_4_.7H_2_O 1 mL, 1 M CaCl_2_.2H_2_O 1 mL, 5 mg/mL cholesterol in ethanol 1 mL, 1 M KPO_4_ buffer 25 mL, and 100 µg/mL penicillin-streptomycin 1 mL were added under the laminar flow cabinet using sterile techniques with a constant mixing to make sure the mixture was homogeneous, and later it was poured into 100 mm diameter Petri plates and left for 24 h under 22 ± 2 °C temperature conditions. After synchronizing, a total of 210 synchronized worms were prepared, and 35 were divided into each plate separately. For this experiment, 15 plates were prepared and divided into five groups, three as the control group and three as the experimental groups. The NGM plates were seeded with 500 µL of *Escherichia coli* OP50-1 strain (OD:0.4; treated at 65 °C for 10 min to kill) under sterile conditions as a food source; the control groups only contained the *E. coli* and a concentration of 75 µM and 150 µM emodin and aloe-emodin was added to the experimental groups. DMSO was included in the control group. The seeded plates were left overnight at room temperature to allow the *E. coli* OP50-1 to grow.

Our experiment used the thermotolerance test that is part of survival assays to evaluate and screen the stress evolution of the experimental worms exposed to emodin and aloe-emodin compared to the control group on worm’s lifespan within hours. The experiment started at the end of the L4 larval stage; the plates were incubated at 35°±0.8 °C for 12 h in an incubator, and images were captured with a high-resolution scanner (Epson Perfection, V800 Photo) starting from T0 the plates were scanned in every 20 min, till every worm dies. The worms that were not moving in two consecutive scans were accepted as dead. The results were scored and analyzed using a statistical method, The Kaplan-Meier, to estimate the survival function.

### Statistical analysis

Statistical analysis was performed using Graphpad Prism 6. Statistical differences of the treatment groups vs. the untreated control group were evaluated with one-way ANOVA followed by the Tukey test. The results were represented as mean ± standard deviation (SD)., *p < 0.05 and **p < 0.01 versus the control group.

## Results

### Intracellular ATP content

**Fig. 2:**
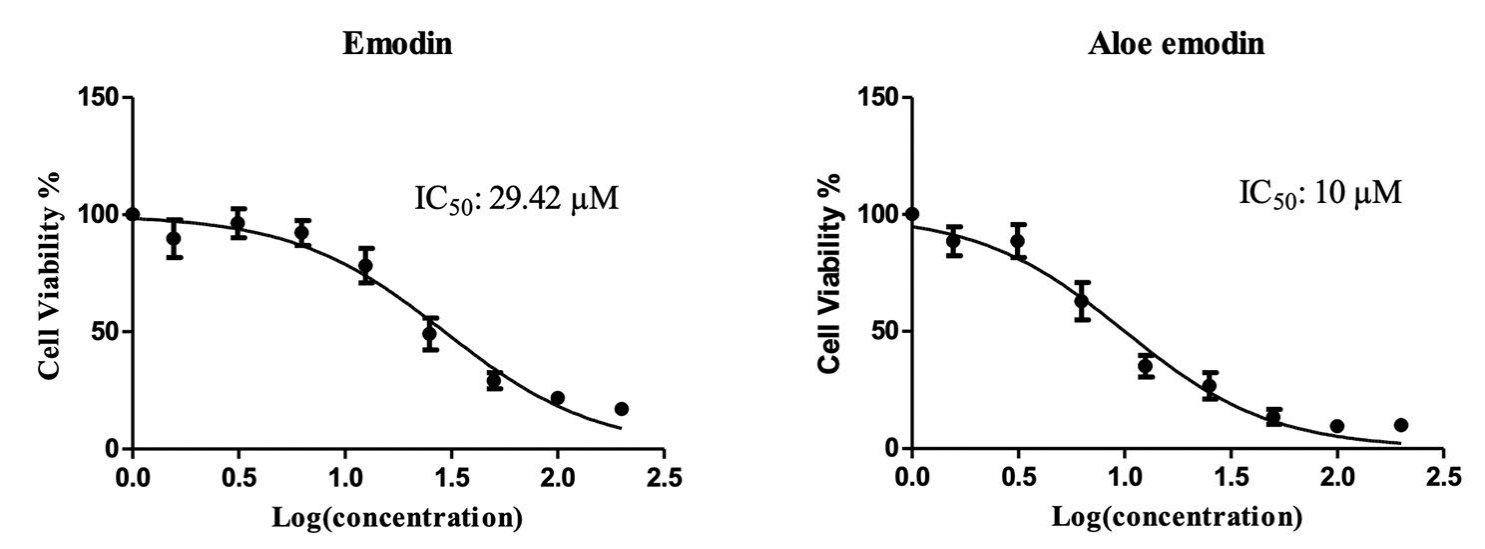
: **Effects of emodin and aloe-emodin on cellular ATP content of CCD-1079Sk skin fibroblasts after 24 h treatment.** The cells were treated with 0-1000 µM concentrations and the IC_50_ values are presented. Data are expressed as mean ± SD

Emodin and aloe-emodin inhibited the ATP content of the CCD-1079Sk skin fibroblast cells after 24 h treatment in a concentration dependent manner. The IC_50_ values are 29.42 µM and 10 µM, respectively (Fig. 2). The ATP content of the cells indicates cell viability.

### In vitro wound healing (scratch) assay

**Fig. 3:**
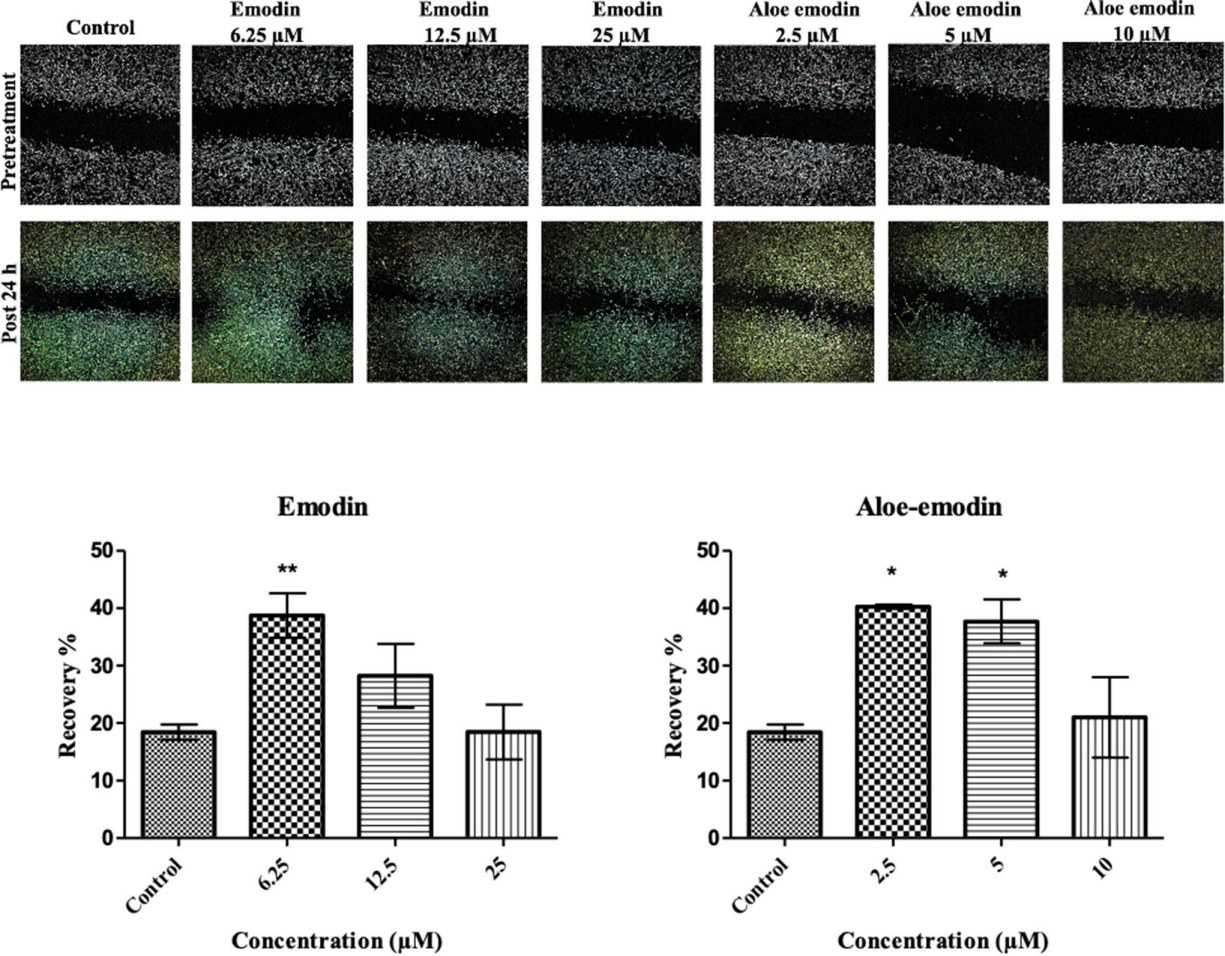
: **Effects of emodin and aloe-emodin on cell migration of CCD-1079Sk skin fibroblasts after 24 h treatment.** The images of the wound areas were taken before and after treatment. The cells were treated with 6.25 µM, 12.5 µM, and 25 µM emodin; 2.5 µM, 5 µM, 10 µM aloe-emodin concentrations. Data are expressed as mean ± SD, *p < 0.05 and **p < 0.01 versus the control group

Emodin and aloe-emodin accelerated cell migration of skin cells after 24 h treatment compared to the control group (Fig. 3). Emodin and aloe-emodin similarly induced cell migration of skin cells in the lower concentration groups (6.25 µM and 12.5 µM concentration groups of emodin, 2.5 µM and 5 µM of aloe-emodin).

### Evaluation of gene expression levels of the MAP kinase pathway

**Fig. 4:**
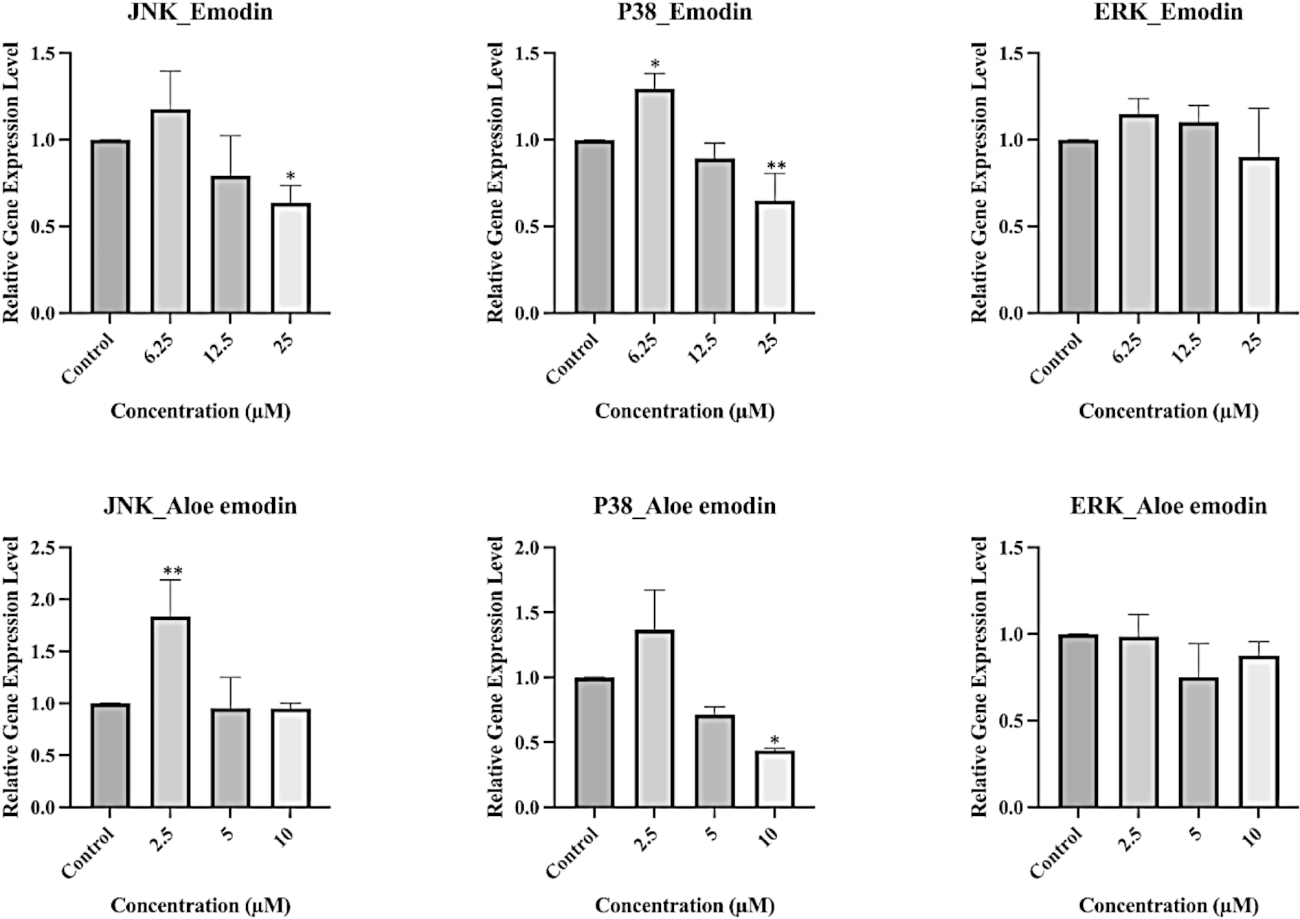
: **Effects of emodin and aloe-emodin on MAP kinase genes (JNK, P38, ERK) after 24 h treatment to CCD-1079Sk skin fibroblasts.** The cells were treated with 6.25 µM, 12.5 µM, and 25 µM emodin; 2.5 µM, 5 µM, 10 µM aloe-emodin concentrations. Data are expressed as mean ± SD, *p < 0.05 and **p < 0.01 versus the control group

*JNK, P38*, and *ERK* gene expression levels of the skin cells were evaluated after 24 h emodin and aloe-emodin treatment (Fig. 4). Emodin induced *JNK* and *P38* at 6.25 µM concentration and showed significant downregulation at 25 µM. The change in the *ERK* level was not significant (p > 0.05). Similarly, aloe-emodin induced *JNK* and *P38* gene expressions in the lowest treatment group (2.5 µM) and showed significant *P38* downregulation at 10 µM. Although the change in the *ERK* level was not significant (p > 0.05), it was observed to be downregulated in the higher concentrations of emodin and aloe-emodin.

### Molecular docking study

**Fig. 5:**
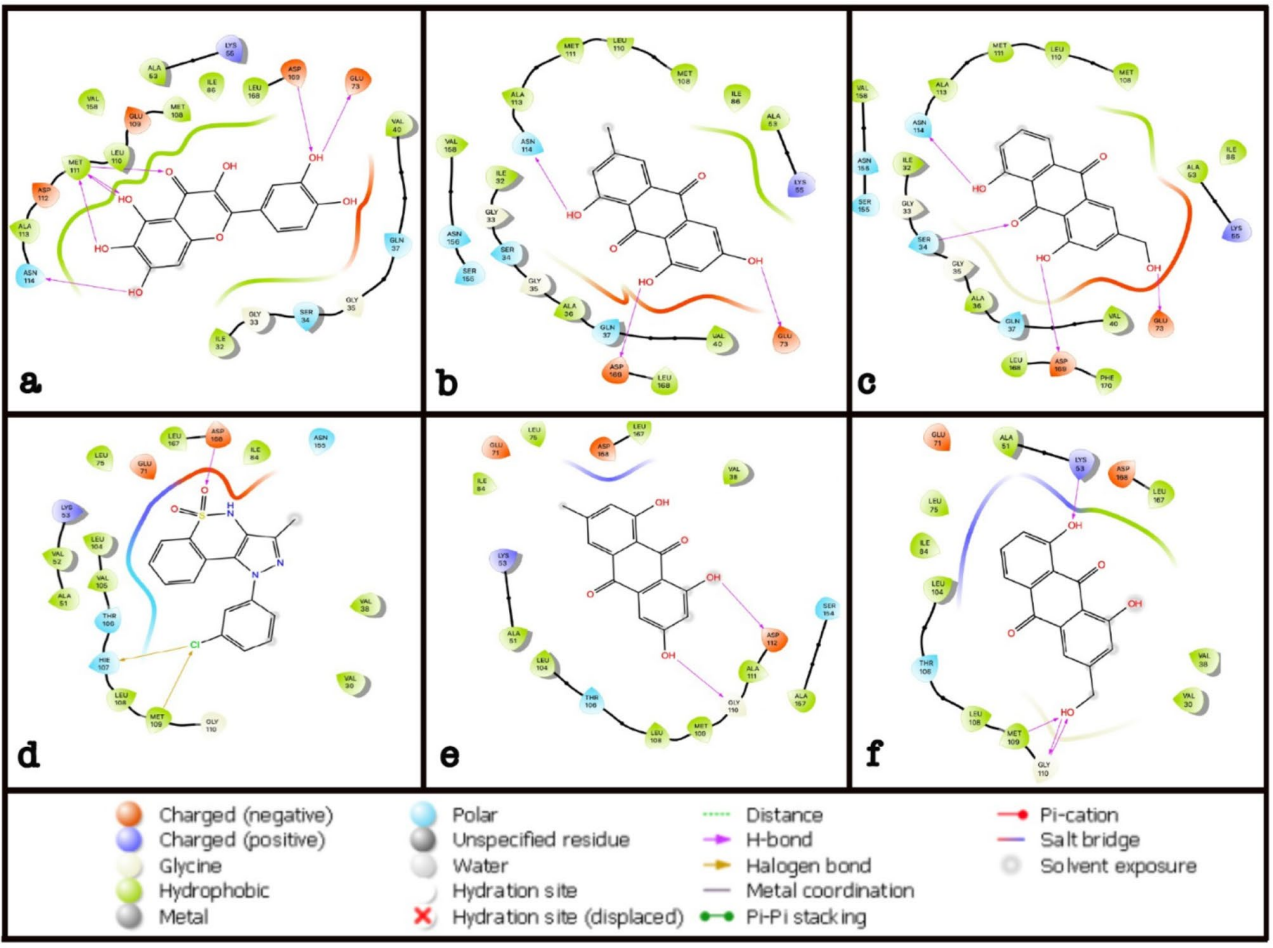
: **2D interactions with the active site of the crystal structure of JNK1 and P38alpha (PDB ID:3V3V, 5OMH). (a)** Quercetagetin and its 2D interactions with the active site of Jun N-terminus kinase-1 (PDB ID: 3V3V), **(b)** Emodin and its 2D interactions with the active site of Jun N-terminus kinase-1 (PDB ID: 3V3V), **(c)** Aloe-emodin and its 2D interactions with the active site of Jun N-terminus kinase-1 (PDB ID: 3V3V), **(d)** 9Y5 and its 2D interactions with the active site of p38alpha (PDB ID: 5OMH), **(e)** Emodin and its 2D interactions with the active site of p38alpha (PDB ID: 5OMH), **(f)** Aloe-emodin and its 2D interactions with the active site of p38alpha (PDB ID: 5OMH). NL: Native ligand

Molecular docking studies were carried out to examine the states of emodin and aloe-emodin in the active site of the enzymes Jun N-terminus kinase-1 (JNK1) and p38alpha. Emodin and aloe-emodin interactions with the binding sites of JNK1 and docking scores are given in Fig. 5; Table 2. In the active site of the JNK1 receptor, there are hydrogen bonds between the hydroxyl groups of emodin with the GLU73, ASN114, ASP169 amino acids. Similarly, there are hydrogen bonds between the hydroxyl groups of aloe-emodin with GLU73, ASN114, ASP169, and a hydrogen bond between one of the carbonyl groups of aloe-emodin with the SER34.

Emodin and aloe-emodin interactions with the binding sites of p38alpha and docking scores are given in Fig. 5; Table 3. In the active site of the p38alpha receptor, there are hydrogen bonds between the hydroxyl groups of emodin with GLY110, ASP112 and there are hydrogen bonds between the hydroxyl groups of aloe-emodin with LYS53, GLY110, MET109.

**Table 2:** The docking scores of the emodin, aloe-emodin and quercetagetin and their interactions with the active site of Jun N-terminus kinase-1 (JNK1) enzyme crystal structure (PDB ID: 3V3V)

Ligand	Docking Score (kcal/mol)	H-bond
**Quercetagetin**	-9.37	GLU73, ASN114, ASP169, MET111
**Emodin**	-8.46	GLU73, ASN114, ASP169
**Aloe-emodin**	-8.76	SER34, GLU73, ASN114, ASP169

**Table 3:** The docking scores of the emodin, aloe-emodin and 9Y5 and their interactions with the active site of p38alpha enzyme crystal structure (PDB ID: 5OMH)

Ligand	Docking Score (kcal/mol)	H-bond	Halogen bond
**9Y5**	-5.9	ASP168	HIE107, MET109
**Emodin**	-6.5	GLY110, ASP112	-
**Aloe-emodin**	-6.16	LYS53, GLY110, MET109	-

**Fig. 6:**
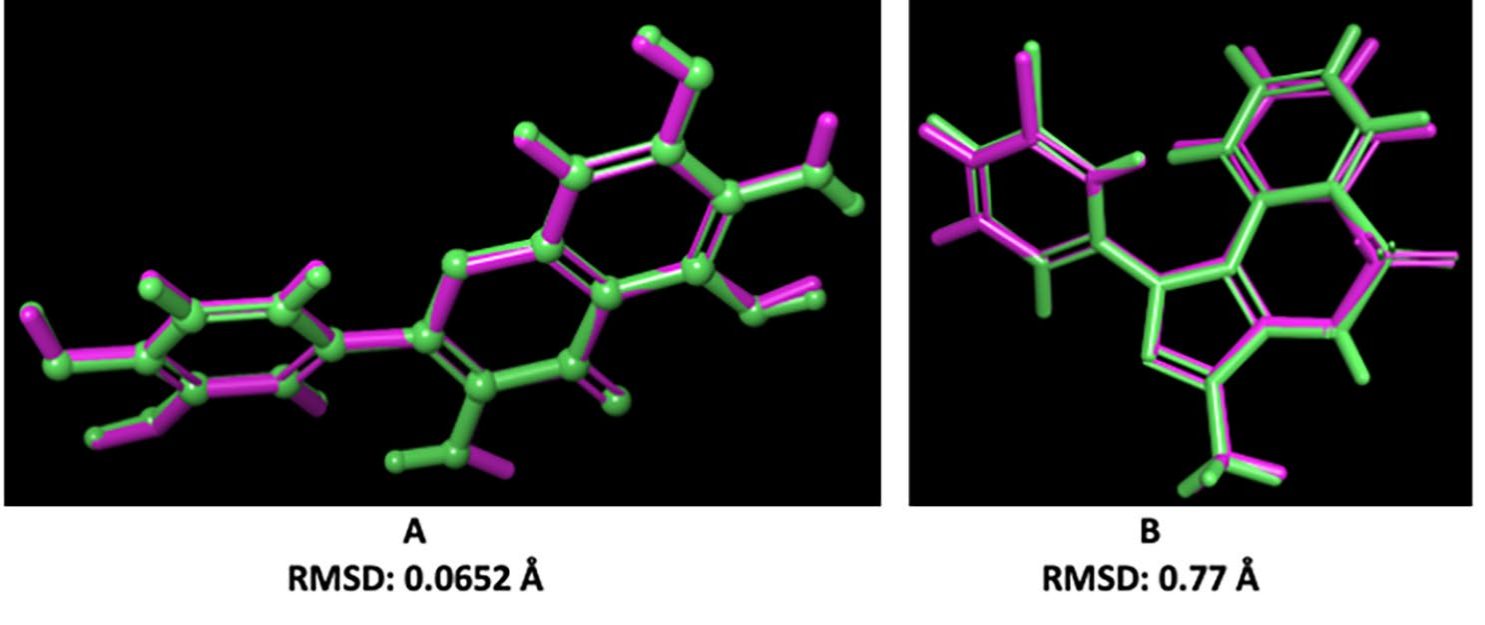
: **Validation of the docking models.** A: Superposition of docked pose (green) and experimental binding conformation (pink) of quercetagetin in the binding pocket of 3V3V. B: Superposition of docked pose (green) and experimental binding conformation (pink) of 9Y5 in the binding pocket of 5OMH

To verify the reliability of the docking study, the co-crystalized ligands were redocked to the active sites of proteins and the root- mean-square deviation (RMSD) was calculated (Fig. 6). Glide successfully reproduced the experimental binding conformations of quercetagetin in the binding site of 3V3V with an acceptable RMSD value 0.0652 Å. Furthermore, the RMSD value was determined as 0.77 Å for 5OMH which was also an acceptable value which indicates the validity of the studied docking model.

### *C. elegans* thermotolerance study

**Fig. 7:**
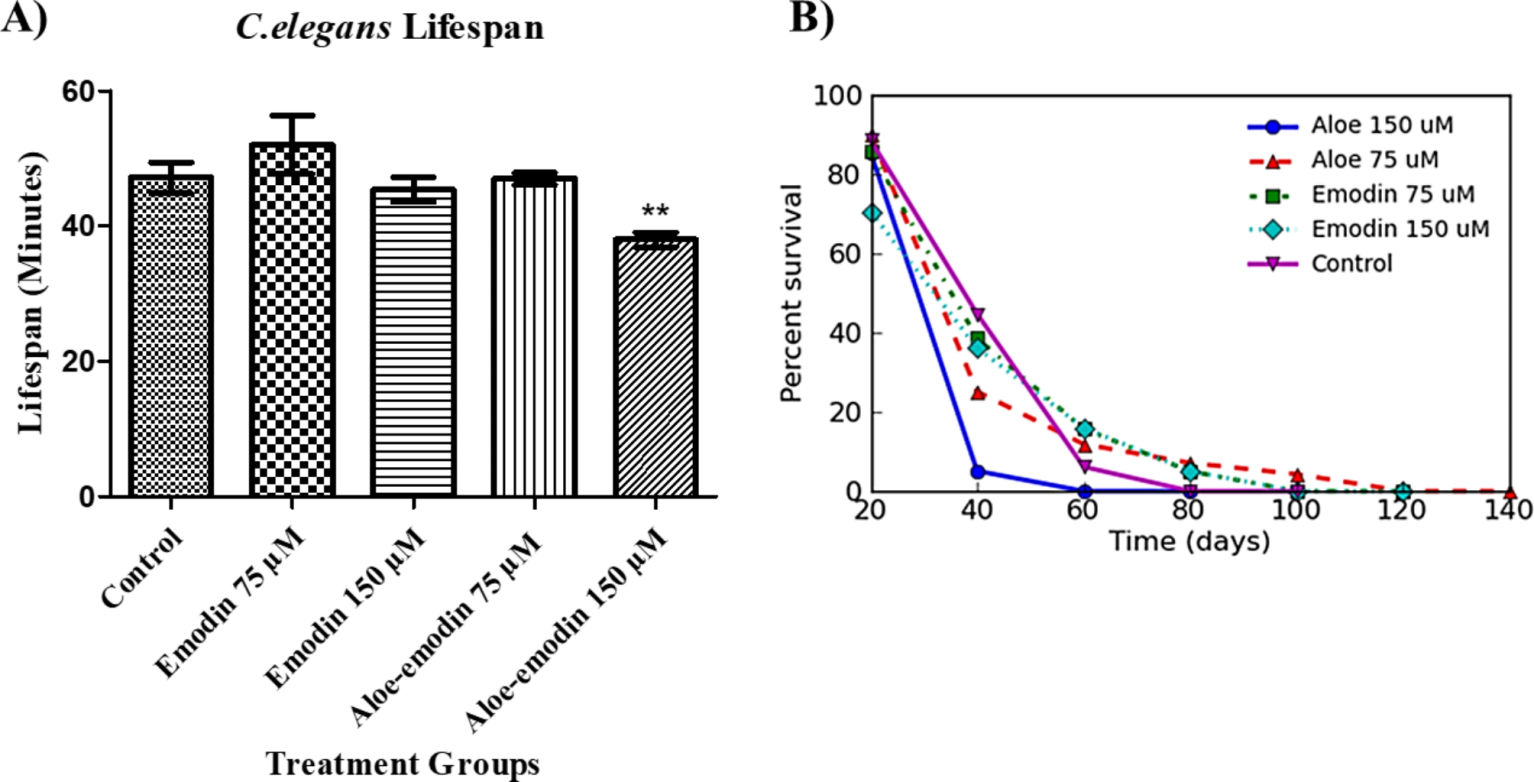
: **Effects of emodin and aloe-emodin on*C.elegans*lifespan (A). Survival / Log Cumulative Hazard Plot (B).** The cells were treated with 75 µM and 150 µM emodin and aloe-emodin concentrations. Data are expressed as mean ± SD, **p < 0.01 versus the control group

The effects of emodin and aloe-emodin on the lifespan of *C.elegans* worms under heat stress are given in Fig. 7., where emodin shows an insignificant (p > 0.05) positive effect in the lower concentration and aloe-emodin shows a significant (p < 0.01) inhibitory effect in the higher concentration, compared to the control group.

## Discussion

In traditional folk medicine, herbs are widely used and natural molecules/derivatives from plants are found to be effective in wound healing. Although the use of natural products in wound healing is common, studies investigating the mechanisms of action are insufficient [4]. Emodin and aloe-emodin which are two anthraquinone derivatives found in the content of plants have positive effects in the wound healing process. The aim of this study is to elucidate the mechanisms of emodin and aloe-emodin comparatively, which are potential agents in wound healing.

This is the first study to evaluate emodin and aloe-emodin’s effects on the human CCD-1079Sk fibroblastic skin cell line. Fibroblasts play an important role in the wound healing process; the fibroblasts of damaged tissue are recognized as the key cells in the proliferative phase of healing by enabling cell proliferation, differentiation and migration. Among these processes, cell migration is a critical biological response during wound healing and allows measurement of wound healing ability by analyzing the migration of cells in vitro [19]. The IC_50_ values of emodin and aloe-emodin against the human fibroblasts were found to be 29.42 µM and 10 µM, respectively. Therefore, aloe-emodin is more potent in terms of cytotoxicity. In a recent study, the molecules’ effects on different skin cell lines were similar showing that aloe-emodin is more potent in growth inhibition compared to emodin [20].

The presence of the hydroxy methyl group in aloe-emodin instead of the hydroxyl group in the C-3 position of emodin, and the absence of the methyl group in aloe-emodin in the C-6 position potentially affected the activities of the compounds. These differences in the functional groups at the C-3 and C-6 positions of the two molecules with the same skeleton structure had significant effect on the potence of the compounds as seen in the *in silico* and in vitro results.

There are current studies using fibroblast cells in in vitro wound healing testing [9, 10]. According to the results obtained from the in vitro scratch assay in our study, low concentrations of both emodin and aloe-emodin accelerate cell migration whilst higher concentrations tend to attenuate the process. Emodin and aloe-emodin was observed to inhibit cell migration in 20 µM concentration in which the HaCaT cell line was used as healthy human skin cells [20]. In an in vitro wound healing study on human fibroblasts, it was shown that emodin is a potent profibrinolytic and wound healing agent [21]. In a study on rats, it was determined that the wounds shrunk significantly after treatments of emodin was applied topically in excisional injuries [22]. Another in vivo study on mice with excision wounds, it was found that wound healing rates were increased and the time to epithelization was decreased in aloe-emodin-treated mice [23].

The MAP kinase family, which is actively involved in wound healing, is a well-characterized large family of serine/threonine kinases and regulates important cellular processes such as proliferation, migration, oncogenesis, differentiation, and inflammation. The MAP kinase family is examined in three major groups: Jun N-terminus kinase (JNK) pathway, extracellular signal regulatory kinases (ERK1/2) pathway and P38 group protein kinase pathway. [5–7]. JNK activity is shown to play an important role in the migration of fibroblasts in wound healing experiments [8]. ERK1/2 is one of the main molecules of the MAPK signaling pathway which has been implicated in cell proliferation and plays a critical role in promoting angiogenesis during wound healing [6]. Likewise, P38 is known to play a role in apoptosis, cell differentiation and cell migration, and furthermore, a strong link has been established between the P38 pathway and inflammation and has been associated with cytokine-induced cell migration [5, 7, 9].

We conducted a molecular docking study to roughly evaluate the binding potential of emodin and aloe-emodin against the proteins JNK and P38 which were significantly affected at the gene level after in vitro treatment with the molecules. The docking study shows that emodin and aloe-emodin may have a great potential to inhibit the proteins giving high docking scores and aloe-emodin was again observed to be more potent compared to emodin against JNK1, and in fact, was even more potent than the positive control against P38. According to the docking studies, JNK1 enzyme inhibition docking score was very close to the native ligand and this may be due to the similarity with the structure with quercetagetin which is an anthraquinone derivative. However, even though the structure-activity relationship with p38 is not in correlation with its native ligand (9Y5) structure, the two molecules’ docking scores were higher than 9Y5. Although preliminary, the results of the in vitro studies are in line with the *in silico* results, the observed *JNK* and *P38* gene levels indicate that aloe-emodin is more potent compared to emodin in enzyme interaction activities along with cell viability and cell migration.

The gene expression level after emodin and aloe-emodin treatment of the fibroblasts supports the dual effect seen in the cell migration study, i.e., *JNK* and *P38* was observed to be induced in the studied lowest concentrations whilst higher concentrations inhibited the gene expression levels. Many phytochemicals induce hormetic responses on physiological functions, i.e., positive effects are seen at low concentrations and negative effects are observed at high concentrations. Emodin is listed among the compounds as a molecule that produces hormetic effects on health and longevity in vivo [24].

Some previous studies also show significant changes after emodin and aloe-emodin [25, 26]. In a study using hepatic stellate cells, P38 activity was observed to be inhibited after emodin treatment in a concentration-dependent matter while JNK was not [27] suggesting emodin as a potential candidate for the treatment of hepatic fibrosis. Similarly, the *P38* expression was inhibited after emodin and aloe-emodin treatments in the high concentration groups in our study. Although the study concentration range is some-what similar to our study, the inducement of *P38* and *JNK* in the lowest concentration group in our study may be due to cell line difference and/or expression analyses in the gene level. In the study of Cui et al., after emodin treatment to HepG2 cell line, P38 phosphorylation was observed to be induced in the protein level while ERK was inhibited, furthermore, JNK level was not observed to change [26]. *P38* and *JNK* expressions were induced in the low concentrations in our study, although *ERK*, on the other hand, seemed not to be affected significantly. This may be because ERK is one of the main molecules of the MAPK signaling pathway activated through the three-component protein kinase cascade Ras → Raf → MEK1/2 and although the cascade may actually be affected ERK may not seem to be affected in the gene level of the cells in our study [28]. Collectively, these results suggest the concentration-dependent effects of the molecules, which should be verified with further in-depth molecular methods and future in vivo studies.

Next, to see the comparative toxic effects of emodin and aloe-emodin in vivo, we used the *C. elegans* nematode model and observed that emodin had little unsignificant effects in the studied concentrations (75 µM and 150 µM), although the lower concentration slightly increased the lifespan. A study conducted with the same model reports extension of the lifespan after 150 µM emodin treatment while 300 µM emodin had little effect, which is in some way in line with our results (lower concentration slightly extended lifespan while higher concentration had no effect) [29]. When we look at aloe-emodin, the high concentration of aloe-emodin shortened the lifespan significantly in our study. This suggests that aloe-emodin should be used in lower concentrations due to its potent activity compared to emodin. In *C. elegans*, thermotolerance assays are preferred for in aging studies because of its short experimental since there is a proven significant correlation between thermotolerance and life span phenotypes in *C. elegans* [30–32].

## Conclusions

In this study we aimed to analyze the mechanistic effects of emodin and aloe-emodin, and we focused on evaluating the related pathways in the CCD-1079Sk human fibroblastic skin cell line. Indeed, the findings should be verified with other cell lines along with further molecular methods, and further *in silico, in vitro, in vivo* studies to conclude the molecules’ wound healing effects. This study provides a great opportunity to better understand the mechanism of the wound healing process, and to compare the two molecules’ biological effects where the only molecular differences between them are the presence of a hydroxy methyl molecule at position C-3 and an absence of a methyl group at C-6 yet showing a significant influence. Overall, aloe-emodin was found to be more potent on cell viability, cell migration, gene expression levels of the MAP kinases in healthy fibroblastic skin cells, and on the lifespan of *C. elegans.* This study contributes to the accumulating studies showing the potential of emodin and aloe-emodin to be used as therapeutic wound healing agents. In conclusion, these results reveal the functional effects and the biological factors that interact in the wound healing process of emodin and aloe-emodin, and give a possible treatment alternative to shorten the duration of wound care.

## Data Availability

The datasets used and/or analyzed during the current study are available from the corresponding author on reasonable request.
